# Use of tobacco, alcohol and cannabis in late adolescence: roles of family living arrangement and socioeconomic group

**DOI:** 10.1186/s12889-020-09476-w

**Published:** 2020-09-04

**Authors:** Myriam Khlat, Océane Van Cleemput, Damien Bricard, Stéphane Legleye

**Affiliations:** 1 Institut National d’études Démographiques (INED), 133 boulevard Davout, 75980 Paris, Cedex 20 France; 2 Centre de Recherche en Démographie (DEMO), Université Catholique de Louvain, Ottignies-Louvain-la-Neuve, Belgium; 3 Institut de Recherche et Documentation en Économie de la Santé (IRDES), Paris, France; 4 INSERM, Mental Health in Public Health, Université Paris-Sud, Université Paris-Saclay, Villejuif, France

**Keywords:** Adolescent, Substance use, Tobacco, Alcohol, Cannabis, Family structure, Socioeconomic group

## Abstract

**Background:**

France has one of the highest levels in Europe for early use of legal and illegal psychoactive substances. We investigate in this country disparities in adolescent problematic substance use by family living arrangement and parental socioeconomic group.

**Methods:**

The data used were from the 2017 nationally-representative ESCAPAD survey, conducted among 17-year-olds in metropolitan France (*N* = 39,115 with 97% response rate). Prevalence ratios (PR) were estimated using modified Poisson regression.

**Results:**

Adolescents living in non-intact families (44%) reported daily smoking, binge drinking and regular cannabis use (respectively ≥3 episodes and ≥ 10 uses in the last 30 days) much more frequently than those living in intact families (for example, the PR estimates for father single parent families were respectively 1.69 (1.55–1.84), 1.29 (1.14–1.45) and 2.31 (1.95–2.74)). Socioeconomic differences across types of families did little to explain the differential use. Distinctive socioeconomic patterns were found: a classical gradient for smoking (PR = 1.34 (1.22–1.47) for the most disadvantaged group relative to the most privileged); an inverse association for binge drinking (PR = 0.72 (0.64–0.81) for the most disadvantaged relative to the most privileged), and no significant variation for cannabis use.

**Conclusion:**

Our findings shed light on the consistency of the excess use of adolescents from non-intact families and on the substance-specific nature of the association with parental socioeconomic group. Preventive approaches at the population level should be complemented by more targeted strategies.

## Introduction

Adolescence is a developmental period of heightened risk-taking behaviours, with substance use and misuse at the forefront of the health concerns. In epidemiological surveys, consumption of tobacco, alcohol and cannabis are appreciable from early adolescence, accelerate in late adolescence and culminate in the early twenties [1]. Analysing the contexts related to the engagement in those behaviours during the course of the transition between adolescence and adulthood is essential for prevention purposes.

Family factors hold a good place as predictors of adolescent substance use [2], with a prominence of two dimensions: family living arrangements and family socioeconomic level. Regarding the former, prior research has provided evidence for a strong association between substance use and not living with both parents, i.e. living in “non-intact families”, be they single parent families or stepparent families. However, the increasing diversity of “non-intact families” is not yet fully explored, and particularly the situation of single father families and that of single-parents sharing custody deserve further attention [3]. On the other hand, findings may be context-specific, as it has been suggested that the rarer a family configuration, the stronger its association with youth consumption [4], and at the opposite the greater the availability of a substance, the stronger its association with the different types of “non-intact families” [5].

Socioeconomic background also exerts an influence on adolescents’ substance use. Among adults, there is a social gradient in health-related behaviours, but the relation between socioeconomic status (SES) and health does change across the lifespan [6, 7]. For adolescents and young adults, variable patterns have been found, depending on substance and study context. Lower family SES has generally been associated with greater prevalence of cigarette smoking, with differences across countries [8–10]. For the other substances, findings are not consistent, with some studies reporting a negative association of cannabis and alcohol consumption with family SES among adolescents [11], and others no clear relation [6]. There is also evidence that adolescents with higher SES may be at risk for developing substance use disorders [1, 12]. Overall, no coherent vision of the social gradient of substance use among adolescents emerges from the literature.

Another important aspect is that family structure and socioeconomic conditions are inter-related in several ways. First, there may be differences in the risk of divorce by occupational class, and in the United States adolescents in lower SES families were found to be less likely to live with both their parents than those from middle and upper-class families [13, 14]. Second, family disruption affects material circumstances of the household. Notably, single-parent families, and particularly single mother families, are more likely to experience economic hardship than intact families and than other types of non-intact families such as stepparent families [4, 15]. Socioeconomic factors may therefore at the same time confound and mediate the relationship of family structure to adolescent substance use. In the literature, the focus was put on one of the two factors, with a mere adjustment on the other, but there is a lack of research fully considering and discussing those inter-related dimensions as independent variables in their own right to explain disparities in adolescent smoking and drinking. Testing for gender-specific effects may also be relevant, as it has been hypothesized that children who live with their same-sex parent are in a better situation than their peers who live with an opposite-sex parent [16], or alternatively that girls may be more affected than boys by disruptions in their childrearing environment [17].

Our aims in this study are to explore disparities in adolescent substance use in France, considering at the same time the full diversity of family living arrangements and the socioeconomic group of the parents. France is currently one of the countries with the highest prevalence levels in Europe for early use of both legal and illegal psychoactive substances, despite improvements in prevention since the early 2000s, in young adults [18, 19] as well as in adolescents aged 15–16 years [20]. While daily tobacco smoking is on the decline, problematic alcohol-related behaviours are a source of concern, and the proportion of problem cannabis users increased in 2017 [21]. On the other hand, the number of divorces has considerably increased in France during the past two decades [15], and diversification of family configurations is well under way [15, 22]. The French context is therefore very well suited to a study of disparities in adolescent substance and the role of family factors.

We use a large-scale nationally representative survey conducted in 2017 in France, providing both family background information and substance use reports from late adolescents. The three most commonly used substances at adolescence are investigated, with a focus on daily smoking, binge drinking and regular cannabis use. Our research questions are:

1. What is the relationship between family living arrangement and use of the three substances, independently of parental socioeconomic group? And reciprocally; What is the relationship between parental socioeconomic group and use of the three substances, independently of family living arrangement?
2. What is the relative importance of family living arrangement vs parental socioeconomic group?

For living arrangement, we compare the different configurations, and particularly the different non-intact types regarding their relation with substance use. We also test for gender-specific effects. Lastly, we assess whether the social pattern of use for the different substances is a classical one, with greater use in the most disadvantaged groups as is found in the literature on adults, or whether it is reversed or non-existent.

## Methods

### Design, population and data collection

This study used data from the latest edition of the french cross-sectional Survey on Health and Substance Use on National Defence and Citizenship Day (Enquête sur la Santé et les Consommations lors de l’Appel de Préparation À la Défense (ESCAPAD)) which is implemented since 2000 by the French Monitoring Centre for Drugs and Drug Addiction (Observatoire Français des Drogues et des Toxicomanies (OFDT)) to monitor the consumption of psychoactive substances by adolescents in France [21].

Renewed every 3 years, the ESCAPAD survey is conducted over a two-week period during the National Defence and Citizenship Day (Journée Défense et Citoyenneté (JDC)), an annual one-day session held by the French Ministry of Defence. At the age of 16, all French nationals are automatically registered to the National and Regional Service Office to be called upon by the army when they turn 17 to participate to a JDC session. The participation certificate delivered at the session is required for registration to the baccalaureate examination, to the driving test and at public universities. The survey consists of a paper-based self-administered questionnaire whose average duration is 20 min. In 2017, 43,892 adolescents were surveyed in metropolitan France and the participation rate was 97.4%. After removal of questionnaires poorly or insufficiently completed and of respondents aged over 18 years, the final sample comprised 39,115 adolescents (49.9% female, age range: 17–18 years, Mean age = 17.41). Descriptive statistics for the final sample are shown in Table 1. Overseas departments are not studied here.

**Table 1:** Sample description (ESCAPAD survey 2017, metropolitan France)

	Males	Females	Total
	Weighted percentages (%)		
**Age**			
17	91.7	90.5	91.1
18	8.30	9.5	8.9
**Daily tobacco smoking** ^**a**^	26.2	23.7	25.0
**Binge drinking** ^**b**^	21.4	10.8	16.2
**Regular cannabis use** ^**c**^	9.5	4.4	7.0
**Family living arrangement**			
Intact family	57.8	56.9	57.4
Parent-stepparent	9.3	10.0	9.6
Father single parent	3.5	2.8	3.1
Mother single parent	14.6	16.1	15.3
Shared custody	6.1	5.6	5.8
Other or unknown	8.9	8.6	8.8
**Parental socioeconomic group**			
Very privileged	7.0	7.4	7.2
Privileged	19.6	19.6	19.6
Intermediate	28.6	29.4	29.0
Modest	34.1	33.5	33.8
Disadvantaged (unknown or no answer)	10.7	10.2	10.5
**Total**	**19,611**	**19,504**	**39,115**

^a^defined as smoking one cigarette (packed or hand-rolled) or more per day

^b^defined as experiencing three risky-single occasions drinking per month or more. Risky single occasion (drinking episode) defined as consuming 5 or more drinks on one single occasion

^c^defined as smoking cannabis 10 times per month or more

### Outcomes measures

Measures of binge drinking, daily tobacco smoking and regular cannabis use were based on the current definitions recommended by the OFDT, which allows a comparison with previous ESCAPAD surveys and other health barometers.

Binge drinking was assessed by the question: “How many times did you have 5 or more drinks in one occasion during the last 30 days?”, following the standard question used in other adolescent surveys (European School Survey Project on Alcohol and Other Drugs (ESPAD), Health Behaviour in School-Aged Children (HBSC)). Answers ranged from none to thirty times or more: we used a binary indicator, retaining three or more episodes during the month before the survey [21] as cutpoint. The non-response rate for this question was 1.1%.

Daily tobacco smoking was defined as the report of a daily use of at least one tobacco cigarette in the past 30 days (yes/no) through the question “during the last 30 days, have you smoked cigarettes?” (never; less than once a week; less than once a day; 1–5 per day; 6–10 per day; 11–20 per day; more than 20 per day). Adolescents consuming at least one cigarette per day were considered as daily smokers. The non-response rate was below 1 % (0.6%).

Regular cannabis use was assessed by the question: “How many times did you consume cannabis during the last 30 days (hashish, weed, marijuana)?”, with seven categories ranging from “never” to “30 times or every day”. We followed the OFDT definition of regular cannabis use as smoking cannabis at least 10 times per month. The non-response rate was 2.3%.

### Demographics and background information

“Family living arrangement” was assessed based on the description provided by adolescents of their relationship with the members of their household. We defined the following categories: “intact family” (living with both parents), parent-stepparent family, father single parent, mother single parent, shared custody (living alternately with mother and father). More complex configurations (such as living with parents and a friend or a partner) and unknowns (only 219 respondents out of 3455 did not provide in detail their family composition, among whom 83 gave no information about their parents) were regrouped in a separate category. No sub-division into mother-stepfather and father-stepmother was made as we observed a large majority of parent-stepparent families of the first type (87%).

The parental socioeconomic group (SES) was categorized by the OFDT based on current socio-professional categories of the parents or, if they were no longer working or dead, on their past occupations [23]. Adolescents with both their parents working as managers, entrepreneurs or skilled tradespeople were assigned to the “very privileged” level and those with only one parent in this type of occupations were assigned to the “privileged” level; those with at least one parent in the group of intermediate professions or farmers were in the intermediate level, and those with at least one parent working as an employee or manual worker were in the modest category. Those not reporting any parental occupation were assigned to the disadvantaged category.

### Analyses

All data analyses were conducted using the SAS statistical software (version 9.4). The independent variables were “Family living arrangement” and “Parental socioeconomic group”, and the outcome variables were “Daily tobacco smoking”, “Binge drinking” and “Regular cannabis use”. Prevalence ratios (PR) and 95% confidence intervals (CI) were computed using modified Poisson regression taking overdispersion into account to yield a robust variance estimation [24]. In the initial step, analyses were run separately with adjustment on age and sex, and in a second step, simultaneously using both independent variables, in addition to age and sex (full model). We also tested for interactive effects of respectively sex and family arrangement and sex and parental socioeconomic group on the three outcome measures. A large majority of the tests turned out not to be significant, and we therefore decided to adjust on sex rather than running separate analyses for males and females.

## Results

Descriptive statistics for the independent variables and substance use outcomes are presented in Table 1. Of all substances, tobacco was the most frequently used, with one adolescent out of four smoking on a daily basis. Binge drinking came next, with about 16% of the adolescents reporting at least three risky single-occasions drinking in the month preceding the survey. Regular cannabis use was rarer, as it concerned 7% of the adolescents. Males had higher prevalence than females, much more so for alcohol and cannabis (two-fold difference).

A small majority of respondents (57%) reported living in an intact family. Among non-intact family living arrangements, mother single parents were the most represented, concerning about 15% of all adolescents, followed by parent-stepparent families (9% of all adolescents), shared custody (6%) and father single parent families (about 4%). The distributions were quite similar for boys and girls, with slightly more girls living in mother single parent families and slightly more boys living in father single parent families.

Table 2 shows the prevalence ratios estimates (PR) associated with the two independent variables in the initial model (separate analyses) and in the full model (combined analyses). For all three substances, boys had higher prevalence than girls, with about a two-fold difference for binge drinking and cannabis. Age also mattered, and 18-year olds were consistently more frequent users than 17-year olds. The results confirm the relevance of family structure, as adolescents living in the different types of non-intact family arrangements had a significantly higher consumption compared to those living in an intact family.

**Table 2:** Regression models for substance use in relation with family living arrangement and parental socioeconomic group (ESCAPAD survey 2017, metropolitan France; PR: Prevalence ratios)

	Daily tobacco smoking		Binge drinking		Regular cannabis use	
	Age and sex-adjusted PR (95% CI)	Full model PR (95% CI)	Age and sex-adjusted PR (95% CI)	Full model PR (95% CI)	Age and sex-adjusted PR (95% CI)	Full model PR (95% CI)
**Females vs Males**	**1.11** (1.07–1.15)	**1.11** (1.07–1.15)	**1.97** (1.88–2.07)	**1.98** (1.88–2.08)	**2.14** (1.98–2.32)	**2.15** (1.98–2.32)
**18 years vs 17 years**	**1.30** (1.23–1.37)	**1.30** (1.23–1.36)	**1.19** (1.11–1.28)	**1.21** (1.12–1.30)	**1.56** (1.41–1.74)	1.58 (1.43–1.75)
**Family living arrangement**						
Intact family	1.0	1.0	1.0	1.0	1.0	1.0
Parent-stepparent	**1.78** (1.69–1.87)	**1.76** (1.67–1.85)	**1.27** (1.18–1.37)	**1.29** (1.20–1.40)	**2.03** (1.81–2.28)	**2.05** (1.83–2.31)
Father single parent	**1.69** (1.55–1.84)	**1.67** (1.54–1.82)	**1.29** (1.14–1.45)	**1.32** (1.17–1.48)	**2.31** (1.95–2.74)	**2.35** (1.98–2.78)
Mother single parent	**1.54** (1.40–1.61)	**1.52** (1.45–1.59)	**1.11** (1.04–1.19)	**1.15** (1.07–1.23)	**2.13** (1.93–2.35)	**2.18** (1.97–2.41)
Shared custody	**1.50** (1.41–1.62)	**1.51** (1.41–1.62)	**1.35** (1.24–1.48)	**1.34** (1.22–1.46)	**1.81** (1.56–2.10)	**1.79** (1.55–2.08)
Other or unknown	**1.83** (1.73–1.93)	**1.81** (1.71–1.91)	**1.55** (1.45–1.67)	**1.59** (1.48–1.71)	**2.41** (2.16–2.69)	**2.45** (2.19–2.74)
**Parental socioeconomic group**						
Very privileged	1.0	1.0	1.0	1.0	1.0	1.0
Privileged	**1.15** (1.05–1.26)	**1.10** (1.01–1.20)	0.97 (0.88–1.07)	0.95 (0.86–1.05)	1.18 (0.99–1.40)	1.11 (0.93–1.32)
Intermediate	**1.23** (1.13–1.34)	**1.16** (1.07–1.26)	0.95 (0.86–1.04)	0.92 (0.84–1.01)	1.17 (0.99–1.38)	1.07 (0.91–1.26)
Modest	**1.31** (1.20–1.42)	**1.20** (1.11–1.31)	**0.86** (0.78–0.94)	**0.83** (0.76–0.91)	1.09 (0.93–1.29)	0.96 (0.82–1.13)
Disadvantaged (unknown or no answer)	**1.34** (1.22–1.47)	**1.19** (1.09–1.31)	**0.72** (0.64–0.81)	**0.68** (0.60–0.76)	1.00 (0.83–1.22)	0.83 (0.68–1.00)

In bold: prevalence ratio estimate statistically different from unity

For socioeconomic group, distinctive gradients were found. Daily smoking followed a classical but moderate gradient, increasing with decreasing socioeconomic group, similar to the pattern in adult populations. At the opposite, the frequency of binge drinking declined with decreasing social status (inverse gradient). For cannabis consumption, no salient differences were found. After adjustment for family structure, the social gradient flattened for smoking, whereas the reversed gradient for binge drinking appeared to be slightly accentuated and a lower cannabis use (with borderline significance) emerged for the most disadvantaged category.

Comparing the three substances, several features emerge. Cannabis stands out as having the strongest associations with non-intact family living arrangement. It is also the least frequently consumed substance, and the only one which use is not socially stratified. The specificity of binge drinking is that it has a non-classical inverse pattern, and has the weakest association with family living arrangement. Smoking is the only substance which follows the classical social gradient, and it is strongly associated with family living arrangement.

In order to contrast more finely the different non-intact family types, we conducted pairwise post-estimation significance testing based on the full model estimates (Table 2). Those tests led us to conclude that adolescents in father single parent families significantly more frequently reported daily smoking than those in mother single parent families or in shared custody, as did those in parent-stepparent families compared to those in shared custody. Adolescents living with their mothers less often reported binge drinking than those living with their fathers or in shared custody. Lastly, adolescents living with only one of their parents were more frequent cannabis consumers than those in shared custody.

## Discussion

Our analyses clearly demonstrate the existence of an intrinsic role of each of the two factors considered: the association of reported use of substances with socioeconomic factors is not explained by the specific distribution of family types within the different levels, and reciprocally, the association with family types is not explained by socioeconomic differences across the different types. Further to that, the association with family types seems stronger than that with socioeconomic level for smoking and cannabis, and at the opposite, the association with socioeconomic level seems stronger than that with family type for binge drinking.

Overall, we confirm that adolescents living in non-intact families are more subject to problematic substance use, compared to adolescents living with both their parents, with little difference between the various non-intact configurations. The observed pattern is very coherent, with strong and consistent associations for tobacco, alcohol and cannabis use.

These results are consistent with a large body of literature that already highlighted that adolescents from non-intact families were more prone to experiment and to regularly use psychoactive substances [3, 25–34]. Several mechanisms have been suggested to explain the association of family structure and adolescent behaviours [35]. First, due to limited parental resources, a lack of supervision or the presence of more permissive rules, adolescents from non-intact families may have lower perception of risk and therefore be more likely to engage in consumption behaviours perceived as problematic. This is a key aspect in sociology of deviance [36]. Second, higher levels of stress are likely within disrupted families, with substance use being resorted to as a coping mechanism for youth when facing this type of adversity. Lastly, socialization models posit the protective role of the interpersonal parent-child ties, which would be closer and stronger in families with two parents.

Over and above this general pattern, there are differences related to the specific family arrangements. Regarding smoking, a study based on data from eleven European countries reported that adolescents living with both parents smoked less than those living with single mothers, and that the latter smoked less than those living with their single fathers, mother-stepfather, or with neither parent [4]. Our findings somewhat accord with this cross-national study, as we find that adolescents living with their mother have the lowest levels of consumption of all non-intact families, but we find additionally that this relative advantage extends to adolescents in shared custody. Further to that, we show that adolescents in parent-stepparent families more frequently report daily smoking than those in shared custody. Overall, those patterns seem to suggest a relative protection from smoking in mother only and shared custody arrangements.

Regarding cannabis and alcohol, in a large cross-national study of data from 37 countries, youth living with both their parents reported less alcohol and cannabis use than those from other types of families [3]. The observed differences were not very large, and the excess consumption was mostly found in father-stepmother families, single father families and grand-parent only families, leading the author to conclude that the absence of the mother may be the common denominator exposing the adolescents to excess substance consumption. Keeping in mind that many of the confidence intervals overlap, our findings tend to corroborate this conclusion for binge drinking, whereas cannabis consumption appears to be more nuanced. Indeed, we found that adolescents in every single type of non-intact families seemed to be at-risk for binge drinking and, to an even greater extent, for cannabis. For binge drinking, the most protective configuration among non-intact families seemed to be the single mother family. For cannabis, adolescents in father single parent as well as those in mother single parent were more frequently cannabis users than those in shared custody, which could be indicative of the importance of the maintenance of the tie with both parents [30].

Another important contribution is that, in the face of prior inconsistent evidence, our analyses reveal distinctive socioeconomic gradients for the three substance uses. We find that daily smoking is the only behaviour which displays the classical type of gradient (higher consumption in adolescents from lower SES families), with however a flatter pattern in the full model. A study based on the 2008 ESCAPAD survey found similar results: higher tobacco use among youth of modest background but higher alcohol and cannabis use in youth from the most affluent families [37]. In a multilevel study spanning 35 countries, adolescents from low affluent families had an increased risk of weekly smoking compared to adolescents from high affluent families [38]. The authors suggested the role of psychosocial risks faced by low affluent adolescents, in terms of their relationships with their parents, living conditions and school achievements, with smoking constituting a coping behaviour in inadequate family environments. Another important aspect is that adolescents with low SES parents are more likely to grow up in contexts where smoking is common and particularly to have role models such as parents or siblings who smoke [39]. Under the influence of stress, negative events and the role modelling to which they are exposed, they may experiment smoking, and, once they do, the addictiveness of nicotine may lead them to shift from occasional to regular use through adolescence and into adulthood [6].

The same rationale cannot however be applied to the other two substances. In a literature review on socioeconomic status and health behaviours in adolescence, low SES was associated with greater cigarette smoking, but had no clear pattern of association with alcohol consumption or cannabis use [6]. In the United States, young adults with the highest family background SES were found to be more prone to alcohol and cannabis use [1]. In a database covering 24 European countries, adolescents in lower socioeconomic positions were found to be more likely to report regular smoking and heavy episodic drinking, but there was no association with recent cannabis use [40]. In France, experimenting with tobacco or cannabis occurred more frequently among youngsters from affluent than from modest or underprivileged families, whereas at the opposite, the latter were more prone to engage in daily or regular use after experimenting [41], and the same applied to binge drinking [42]. We find binge drinking to be more common in the upper SES families, a pattern which may be related to the hypothesis that high SES adolescents engage in negative health behaviours due to the combination of high achievement pressures, increased access to spending money, less adult supervision and permissive parental attitudes [12], or to the isolation from parents very involved in their demanding careers [1]. Lastly, no visible socioeconomic gradient emerges in our study for cannabis use, and for some authors this lack of association may be interpreted as a reflection of the influence of peers and school environments, outweighing the impact of family SES [43].

### Limitations

The ESCAPAD survey, given its magnitude and large sample, supplied powerful measures potentially exploitable at different types and sub-types of families. Yet, some limitations are to be noted. First, a confusion between parents and stepparents appeared when interpreting specific parental situations, likely because no distinction was made in the questionnaire. We suggest this nuance should be added in the next editions of the ESCAPAD project. Second, significant non-response rates were observed for socioeconomic variables (probably due to the adolescents’ lack of knowledge or the difficulty to classify the parental occupation) which consequently inflated the lowest socioeconomic categories. However, no distinctive or highly significant association was observed for this category.

### Policy implications

Our joint analysis of family structure and socioeconomic profile provides original findings on the intrinsic roles of those two major determinants of adolescent substance use. Family structure is at the forefront, as living in a non-intact family is associated with much higher levels of consumption for all three substances. Socioeconomic factors are less influential, and we find distinctive patterns for the different substances. The strength and the consistency of the association of family structure with the different substances is noteworthy, in a context where non-intact families have become almost the norm [15] and where the level of drug use is high [18, 19].

It is therefore important from a public policy perspective, to both conduct prevention at the population level, and build more targeted strategies, as the two approaches are complementary. As adolescents from low SES families are more likely to smoke regularly, they could benefit from targeted interventions through school-based or other institution-based prevention programmes, as suggested by Henkel & Zemlin [8]**.** Tobacco-prevention skill-building programs could be led by teachers, adult facilitators or health professionals, and combine social competence training with training in skills to manage peer pressure [44]. Programs to prevent heavy episodic drinking and cannabis use may be useful in high SES social backgrounds, particularly to develop awareness of the actual adolescent behaviours and encourage the parents to enforce clear rules surrounding substance use [1]**.** As for the risks faced by adolescents after family disruption, prior research has pinpointed the importance of the qualitative aspects of family life, advising that “programs concerned with youth substance use take a holistic approach that includes families, and peers, and mental health problems” [3].

## Data Availability

data are available upon request from Stanislas Spilka (stanislas.spilka@ofdt.fr), Coordinator of surveys and statistical analyses at the French Monitoring Centre for Drugs and Drug Addiction (OFDT).

## Abbreviations

CI: Confidence intervals
DEMO: Centre de Recherche en Démographie, Université Catholique de Louvain, Belgique (Center for Demographic Research, Catholic University of Louvain)
ESCAPAD: Enquête sur la santé et les Consommations lors de l’Appel de Préparation à la Défense (Survey on Health and Use on National Defence and Citizenship Day)
ESPAD: European School Survey Project on Alcohol and Other Drugs
HSBC: Health Behaviour in School-Aged Children
INED: Institut National d’Etudes Démographiques (French Institute for Demographic Studies)
INSERM: Institut National de la Santé et de la Recherche Médicale (French Institute of Health and Medical Research)
IRDES: Institut de Recherche et Documentation en Economie de la Santé (Institute for Research and Information in Health Economics (IRDES))
JDC: Journée Défense et Citoyenneté (National Defence and Citizenship Day)
OFDT: Observatoire Français des Drogues et des Toxicomanies (French Monitoring Centre for Drugs and Drug Addiction)
SES: Socioeconomic status
PR: Prevalence ratios

## References

[1] Patrick ME, Wightman P, Schoeni RF, Schulenberg JE (2012). Socioeconomic Status and Substance Use Among Young Adults: A Comparison Across Constructs and Drugs. J Stud Alcohol Drugs.

[2] Wills TA, Yaeger AM (2003). Family Factors and Adolescent Substance Use: Models and Mechanisms. Curr Dir Psychol Sci.

[3] Hoffmann JP (2017). Family Structure and Adolescent Substance Use: An International Perspective. Subst Use Misuse.

[4] Bjarnason T, Davidaviciene AG, Miller P, Nociar A, Pavlakis A, Stergar E (2003). Family structure and adolescent cigarette smoking in eleven European countries. Addiction.

[5] Bjarnason T, Andersson B, Choquet M, Elekes Z, Morgan M, Rapinett G (2003). Alcohol culture, family structure and adolescent alcohol use: multilevel modeling of frequency of heavy drinking among 15–16 year old students in 11 European countries. J Stud Alcohol.

[6] Hanson MD, Chen E (2007). Socioeconomic Status and Health Behaviors in Adolescence: A Review of the Literature. J Behav Med.

[7] West P (1997). Health inequalities in the early years: is there equalisation in youth?. Soc Sci Med 1982.

[8] Henkel D, Zemlin U (2016). Social inequality and Substance Use and problematic gambling among adolescents and young Adults: a review of epidemiological surveys in Germany. Curr Drug Abuse Rev.

[9] Schaap MM, van Agt HME, Kunst AE (2008). Identification of socioeconomic groups at increased risk for smoking in European countries: looking beyond educational level. Nicotine Tob Res.

[10] Richter M. Risk behaviour in adolescence: Patterns, determinants and consequences [Internet]: VS Verlag für Sozialwissenschaften; 2010. p. 1. Available from: 10.1007/978-3-531-92364-2.

[11] Lemstra M, Bennett NR, Neudorf C, Kunst A, Nannapaneni U, Warren LM (2008). A meta-analysis of marijuana and alcohol use by socio-economic status in adolescents aged 10-15 years. Can J Public Health.

[12] Luthar S, Latendresse S (2005). Children of the Affluent Challenges to Well-Being. Curr Dir Psychol Sci.

[13] Jalovaara M (2000). Divorce by family composition and socioeconomic status in Finnish first marriages. Finn Yearb Popul Res.

[14] Wilcox WB, Wang W. The marriage divide. How and why working-class families are more fragile today: Research brief for Opportunity America-AEI-Brookings Working Class group; 2017. p. 19. Available from: https://ifstudies.org/blog/the-marriage-divide-how-and-why-working-class-families-are-more-fragile-today.

[15] Institut national de la statistique et des études économiques (INSEE) (2015). Couples et familles [Internet].

[16] Powell B, Downey DB (1997). Living in Single-Parent Households: An Investigation of the Same-Sex Hypothesis. Am Sociol Rev.

[17] Kroneman LM, Loeber R, Hipwell AE, Koot HM (2009). Girls’ Disruptive Behavior and its Relationship to Family Functioning: A Review. J Child Fam Stud.

[18] European Monitoring Centre for Drugs and Drug Addiction (EMCDDA) (2012). The state of the drugs problem in Europe [Internet].

[19] European Monitoring Centre for Drugs and Drug Addiction (EMCDDA) (2019). European Drug Report. Trends and Developments. [Internet].

[20] ESPAD Group (2016). ESPAD report 2015: results from the European school survey project on alcohol and other drugs [Internet].

[21] Spilka S, Le Nézet O, Janssen E, Brissot A, Philippon A, Shah J (2018). Les drogues à 17 ans : analyse de l’enquête ESCAPAD 2017. Tendance.

[22] Sobotka T, Toulemon L (2008). Overview Chapter 4: Changing family and partnership behaviour: Common trends and persistent diversity across Europe. Demogr Res.

[23] Spilka S, Ngantcha Beck F, Le Nézet O (2015). Les drogues à 17 ans : analyse de l’enquête ESCAPAD 2014. Tendance.

[24] Zou GY, Donner A (2013). Extension of the modified Poisson regression model to prospective studies with correlated binary data. Stat Methods Med Res.

[25] Schwartz SJ, Zamboanga BL, Ravert RD, Kim SY, Weisskirch RS, Williams MK (2009). Perceived Parental Relationships and Health-Risk Behaviors in College-Attending Emerging Adults. J Marriage Fam.

[26] Surkan PJ, Fielding-Miller R, Melchior M (2012). Parental relationship satisfaction in French young adults associated with alcohol abuse and dependence. Addict Behav.

[27] Kaynak O, Meyers K, Caldeira KM, Vincent KB, Winters KC, Arria AM (2013). Relationships among parental monitoring and sensation seeking on the development of substance use disorder among college students. Addict Behav.

[28] Vanassche S, Sodermans AK, Matthijs K, Swicegood G (2014). The Effects of Family Type, Family Relationships and Parental Role Models on Delinquency and Alcohol Use Among Flemish Adolescents. J Child Fam Stud.

[29] Hemovich V, Crano WD (2009). Family Structure and Adolescent Drug Use: An Exploration of Single-Parent Families. Subst Use Misuse.

[30] Carlsund A, Eriksson U, Löfstedt P, Sellström E (2013). Risk behaviour in Swedish adolescents: is shared physical custody after divorce a risk or a protective factor?. Eur J Public Health.

[31] Gustavsen GW, Nayga RM, Wu X (2016). Effects of Parental Divorce on Teenage Children’s Risk Behaviors: Incidence and Persistence. J Fam Econ Issues.

[32] Jackson KM, Rogers ML, Sartor CE (2016). Parental divorce and initiation of alcohol use in early adolescence. Psychol Addict Behav J Soc Psychol Addict Behav.

[33] Jeynes WH (2001). The Effects of Recent Parental Divorce on Their Children’s Consumption of Alcohol. J Youth Adolesc.

[34] Barrett AE, Turner RJ (2006). Family structure and substance use problems in adolescence and early adulthood: examining explanations for the relationship. Addiction.

[35] Thomson E, McLanahan SS (2012). Reflections on “Family Structure and Child Well-Being: Economic Resources vs. Parental Socialization”. Soc Forces Sci Medium Soc Study Interpret.

[36] Osgood DW, Wilson JK, O’Malley PM, Bachman JG, Johnston LD (1996). Routine activities and individual deviant behavior. Am Sociol Rev.

[37] Legleye S, Spilka S, Le Nézet O, Laffiteau C (2009). Les drogues à 17 ans - Résultats de l’enquête Escapad 2008 [Drug Use of the 17 Years Old - Results of the 2008 Escapad Survey]. Tendances.

[38] Moor I, Rathmann K, Lenzi M, Pförtner T-K, Nagelhout GE, de Looze M (2015). Socioeconomic inequalities in adolescent smoking across 35 countries: a multilevel analysis of the role of family, school and peers. Eur J Public Health.

[39] Kalesan B, Stine J, Alberg AJ (2006). The joint influence of parental modeling and positive parental concern on cigarette smoking in middle and high school students. J Sch Health.

[40] Shackleton N, Milne BJ, Jerrim J (2019). Socioeconomic inequalities in Adolescent Substance Use: evidence from twenty-four European countries. Subst Use Misuse.

[41] Legleye S, Janssen E, Beck F, Chau N, Khlat M (2011). Social gradient in initiation and transition to daily use of tobacco and cannabis during adolescence: a retrospective cohort study: Tobacco and cannabis use during adolescence. Addiction.

[42] Legleye S, Janssen E, Spilka S, Le Nézet O, Chau N, Beck F (2013). Opposite social gradient for alcohol use and misuse among French adolescents. Int J Drug Policy.

[43] West P, Sweeting H (2004). Evidence on equalisation in health in youth from the West of Scotland. Soc Sci Med 1982.

[44] Thomas RE, McLellan J, Perera R (2015). Effectiveness of school-based smoking prevention curricula: systematic review and meta-analysis. BMJ Open.

